# Bioinspired membrane-fusogenic nanomicelles for synergistic chemotherapy, photodynamic therapy, and gas therapy of breast cancer

**DOI:** 10.1016/j.mtbio.2025.102681

**Published:** 2025-12-13

**Authors:** Nan Li, Fengyun Xu, Wei Zhang, Wenke Zhang

**Affiliations:** a State Key Laboratory of Supramolecular Structure and Materials, College of Chemistry, Jilin University, Changchun, 130012, PR China; b Center for Supramolecular Chemical Biology, College of Chemistry, Jilin University, Changchun, 130012, PR China

**Keywords:** Membrane fusion, ELP, Nanomicelle, Synergistic therapy, Breast cancer

## Abstract

Breast cancer remains the most prevalent malignancy globally, posing significant therapeutic challenges. Although nanodelivery systems offer promising strategies for breast cancer therapy, their clinical translation is hindered by critical limitations, including suboptimal biocompatibility, rapid immune clearance, poor targeting specificity, inefficient cellular uptake, and inadequate endolysosomal escape. To overcome these barriers, a cancer cell membrane-coated elastin polypeptide (ELP)-based nanomicelle was designed. This nanomicelle intercalated the photosensitizer IR780 within its hydrophobic region of the cell membrane coating, while encapsulating both rapamycin (Rapa)-loaded ELP micelles and free L-arginine in the hydrophilic core. Benefiting from the homotypic membrane fusion capacity of the cell membrane coating, the nanomicelles enabled active targeting of breast cancer, anchoring IR780 to the breast cancer cell membrane, while releasing L-Arg and Rapa-loaded ELP micelles into the cytoplasm. Under NIR irradiation, IR780 triggered photodynamic therapy (PDT), generating reactive oxygen species (ROS) that simultaneously damaged tumor cell membranes and catalyzed L-Arg conversion to antitumor nitric oxide (NO) gas. Simultaneously, intracellular glutathione cleaved disulfide bonds in the corona of ELP micelles, enabling controlled Rapa release for chemotherapy. In vivo studies demonstrated potent antitumor efficacy of our nanomicelles, including a tumor weight suppression rate of 87.7 %, extensive necrosis, severe DNA fragmentation, and near-elimination of Ki-67 proliferation markers. This work establishes a cell membrane-camouflaged platform for synergistic chemotherapy, PDT, and gas therapy against breast cancer.

## Highlights

• The cancer cell membrane coating enabled IR780 anchoring to cell membrane and cytosolic payload delivery.
• During PDT, IR 780 generates ROS to damage cell membranes and catalyzes the conversion of L-Arg into NO.
• Intracellular GSH cleaves the disulfide bonds of ELP/Rapa micelles, triggering rapamycin release.
• Synergistic chemo-, photodynamic, and gas therapy achieved 87.7% tumor suppression.

## Graphical abstract

**Figure:**
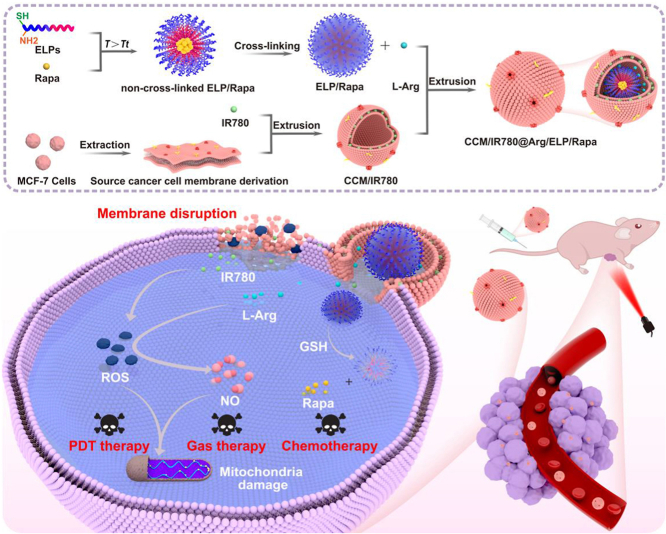

## 1 Introduction

Breast cancer remains the second leading cause of cancer-related mortality among women globally, despite significant advancements in diagnosis and treatment strategies [1,2]. Nanoparticles, which can exhibit robust therapeutic loading capacity, prolonged blood circulation, improved penetration through biological barriers, and enhanced tumor targeting, have emerged as promising approaches for breast cancer treatment [[3], [4], [5], [6]]. In particular, polymeric micelles, with nanometer sized spherical structures possessing a hydrophobic core and a hydrophilic corona, have attracted tremendous attention due to their unique properties [7,8]. Micellization can occur spontaneously in an aqueous environment through the self-assembly of amphiphilic molecules with concentrations above the critical micellar concentration (CMC) [9]. This robust fabrication method facilitates scalable production and clinical translation of polymeric micelles. Furthermore, the hydrophobic core of polymeric micelles can encapsulate hydrophobic breast cancer chemotherapeutics (e.g., paclitaxel, doxorubicin, and docetaxel), thereby addressing their critical limitations associated with poor solubility and low bioavailability [10]. Nevertheless, the potential cytotoxicity of the synthetic amphiphilic polymers for the fabrication of micelles is still a concern, restricting their clinical applications.

Natural polymers have been widely used to fabricate micelles for drug delivery applications due to their superior biocompatibility over synthetic polymers [11]. Particularly, elastin-like polypeptides (ELPs), composed of repeating Val-Pro-Gly-X-Gly pentapeptides (X could be any amino acid except proline), have emerged as an attractive material due to their unique thermal responsiveness [12,13]. ELPs can keep in a monomeric state in an aqueous solution below the transition temperature (*T*_t_), but undergo hydrophobic aggregation when heated above *T*_t_. Notably, the properties of ELPs including *T*_t_ and hydrophilicity/hydrophobicity can be tuned on demand through precise control over their gene sequences. For instance, when ELPs are genetically engineered as diblock polypeptides with *T*_t_ below the physiological temperature, they can spontaneously assemble into micelles in an aqueous environment at 37 °C. This strategy enables both hydrophobic chemotherapeutics encapsulation in the core and functional molecules conjugation on the corona [[14], [15], [16]]. Further stabilization of ELP micellar structures can be achieved by covalent cross-linking after introducing crosslinkable residues (e.g., cysteines or lysines) into ELPs [17]. However, like most nanoparticles, ELP-based micelles are predominantly internalized into cells through endocytosis, leading to entrapment in endosomal-lysosomal compartments. Unfortunately, less than 2 % of the entrapped nanoparticles can successfully escape these degradative organelles, severely compromising their therapeutic efficacy [18].

In biological systems, membrane fusion, the fusion of membrane components between cells, has been considered to play an important role in intercellular communication and material transport. During membrane fusion, the donor cell is initially tethered to the targeted cell via membrane contact, ultimately leading to the formation of a continuous, merged lipid bilayer. Consequently, membrane components are blended while the inner contents of the donor cell are transported directly into the cytosol of the targeted cell [19,20]. Inspired by this natural phenomenon, fusogenic cell membranes have been utilized to coat the nanoparticles for drug delivery applications. This biomimetic approach allows the coated nanoparticles to bypass the conventional endocytic pathway, achieving direct cytosolic delivery and improving intracellular transport efficiency [21]. Meanwhile, the lipid diffusion and mixing during membrane fusion enable the engineering of targeted cell membranes. Moreover, the inherited biological properties of source cells including immune evasion, prolonged circulation, and tissue targeting can be conferred to the coated nanoparticles, thereby enhancing their pharmacokinetic profiles. These unique characteristics make fusogenic cell membrane-coated nanoparticles a promising platform for advanced drug delivery applications.

**Scheme 1:**
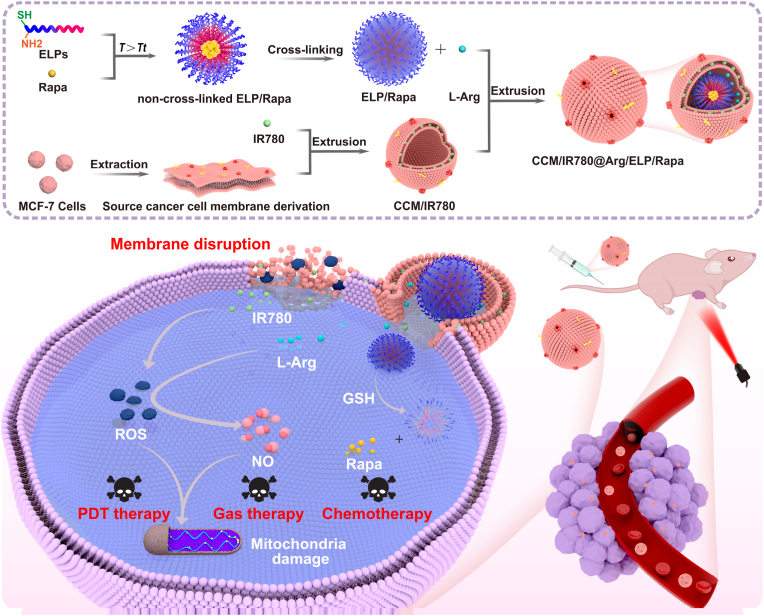
Schematic illustration of CCM/IR780@Arg/ELP/Rapa preparation and membrane fusion, antitumor mechanisms involved after light irradiation.

Inspired by the natural membrane fusion process, we designed a membrane-fusogenic ELP-based nanomicelle to enhance chemotherapy, PDT [22], and gas therapy [23] of breast cancer (Scheme 1). The nanomicelles were fabricated by encapsulating photosensitizer IR780, L-arginine (L-Arg), and rapamycin (Rapa)-loaded ELP micelles into the cancer cell membrane (CCM), which was derived from MCF-7 cells, a human breast cancer cell line possessing intrinsic homotypic fusion capability [21]. To enhance the stability of the ELP micelles during storage and delivery, the micelles were crosslinked via disulfide bonds, which can be broken under reductive environment (e.g., in the presence of intracellular glutathione, GSH). The as-prepared CCM-coated ELP-based nanomicelles can target breast cancer tissues through homologous membrane fusion, which resulted in anchoring IR780 in the cancer cell membranes while directly releasing L-Arg and Rapa-loaded ELP micelles into the cytoplasm (Scheme 1). Upon near-infrared (NIR) irradiation, the membrane-anchored IR780 triggered PDT that simultaneously destroyed cancer cell membranes and catalyzed L-Arg conversion to tumor-suppressive NO gas. Concurrently, the excess GSH [24] cleaved the disulfide bonds in the Rapa-loaded ELP micelles, triggering controlled Rapa release for enhanced chemotherapy. Furthermore, Rapa functions as an mTOR inhibitor to disrupt the pro-survival signals activated by PDT-induced oxidative stress [25]. Meanwhile, both Rapa and PDT-generated ROS can trigger autophagy, synergistically enhancing therapeutic efficacy [26]. Based on these collective beneficial properties, the CCM-coated ELP-based nanomicelles exhibited remarkable therapeutic efficacy in the unilateral breast cancer mice model, demonstrating an 87.7 % tumor weight inhibition accompanied by characteristic DNA fragmentation and significantly reduced proliferation markers (Ki-67) in residual tumors after 14-day treatment. Considering these attractive advantages, our CCM-coated ELP-based nanomicelles show high potential for practical clinical applications for breast cancer treatment.

## 2 Experimental section

### 2.1 Materials and reagents

The pET-*ELP* plasmid was purchased from GENEWIZ (Suzhou, China). Luria-Bertani (LB) broth, agar, DH5α™, and BL21 (DE3) competent cells were from Dingguo Changsheng Biotechnology (Beijing, China). L-Glutathione reduced (GSH), polyethylenimine (PEI), and isopropyl-beta-D-thiogalactoside (IPTG) were from Sigma-Aldrich (Shanghai, China). 1,2-Ethanediyl Bismethanethiosulfonate (MTS-2-MTS), Sulfo-Cy5 NHS ester, IR780, tris (2-carboxyethyl) phosphine hydrochloride (TCEP), and L-Arg were from Aladdin (Shanghai, China). BCA Protein Assay Kit, Membrane and Cytosol Protein Extraction Kit, TBE, 30 % Acr-Bis, N,N,N′,N′-tetramethylethylenediamine (TEMED), Reactive Oxygen Species Assay Kit, DAF-FM DA, 5,5′,6,6′-Tetrachloro-1,1′,3,3′-tetraethyl-imidacarbocyanine iodide (JC-1), Radio Immunoprecipitation Assay lysis buffers (RIPA lysis buffers), and Calcein/PI Live/Dead Assay Kit were purchased from Beyotime Biotechnology (Shanghai, China). Dulbecco's modified Eagle's medium (DMEM), penicillin-streptomycin, trypsin, phenylmethanesulfonyl fluoride (PMSF), phosphate buffered solution (PBS), 4′-6-diamidino-2-pheny-lindole (DAPI), dimethyl sulfoxide (DMSO), ampicillin, and 4 % paraformaldehyde were supplied by Solarbio (Beijing, China). CCK-8 was supplied by Bioss (Beijing, China). MCF-7 cell line was from Pricella (Wuhan, China). Fetal bovine serum (FBS) was from Kangyuan Biotech (Tianjin, China).

### 2.2 Preparation of ELP-based micelles

Before the preparation of the ELP-based micelles, the diblock ELPs containing two cysteines and four lysines at the end of the hydrophilic domains were obtained following published protocols [17,27]. In brief, the diblock ELPs were overexpressed in BL21 (DE3) *E. coli* cells under the induction of IPTG and then purification through inverse transition cycling (ITC). The ICG and Cy5 labeled diblock ELPs were obtained through the modification of the diblock ELPs with the NHS-ICG and NHS-Cy5 followed by purification through ITC, respectively. All polypeptides were examined by 12 % SDS-PAGE and stored at −20 °C in 15 % glycerol.

For the preparation of the non-cross-linked ELP micelles (referred to as non-cross-linked ELP), 0.2 mg/mL of diblock ELPs were incubated at 37 °C for 10 min. To prepare the 1,8-ANS-loaded non-cross-linked ELP micelles (referred to as non-cross-linked ELP/1,8-ANS), 5 μL of 1 mM 1,8-ANS was added to 200 μL PBS containing 0.2 mg/mL diblock ELPs, followed by 10 min incubation at 37 °C.

The cross-linked ELP micelles (referred to as ELP) were prepared by mixing 1 mL of 0.2 mg/mL diblock ELPs and 1 μL of DMF containing 30 mg/mL MTS-2-MTS, followed by 30 min incubation at 37 °C. The ICG and Cy5 labeled cross-linked ELP micelles (referred to as ELP-ICG and ELP-Cy5) were fabricated as described above with the ICG and Cy5 labeled diblock ELPs. The cross-linked ELP micelles (referred to as ELP) were also prepared as a control. All cross-linked micelles were supplemented with 0.5 % (w/v) L-Arg for future preparation of the CCM-coated nanomicelles.

To obtain the Rapa-loaded cross-linked ELP micelles (referred to as ELP/Rapa), a thin-film hydration method was then used. 0.2 mg/mL ELP in 3 mL PBS and 50 μM Rapa in 7 mL acetonitrile were placed into a 100 mL round-bottom flask and loaded onto a rotating evaporator. The mixture was incubated at 40 °C, 120 rpm for 10 min and then dried at room temperature (RT) overnight for the formation of a thin film on the wall of the round-bottom flask. 3 mL H_2_O was added into the round-bottom flask, followed by incubation at 37 °C, 120 rpm for 20 min to obtain the drug-loaded micelles through hydration of the thin film. The unloaded Rapa can precipitate instantly and be removed by centrifugation at 10,000 rpm, 37 °C for 10 min. The micelles were further incubated at 37 °C for 0.5 h in the presence of 0.03 mg/mL MTS-2-MTS to induce the cross-linking reaction. L-Arg was then added to the micelle solution to a final concentration of 0.5 % (w/v) for further use.

### 2.3 Extraction of CCM

The CCM was isolated from MCF-7 cells using a commercial membrane protein extraction kit. Briefly, around 10^8^ cells were suspended in 2 mL of membrane protein extraction buffer containing 1 mM PMSF, cooled on ice for 15 min, and then lysed by 3 cycles of freezing-thawing. Subsequently, the lysate was centrifuged at 700 g, 4 °C for 10 min to remove nuclei and cellular debris. The harvested supernatant was further centrifuged at 14,000 g, 4 °C for 30 min to collect the CCM fraction. The as-prepared CCM was mixed with RIPA lysis buffers to verify the membrane protein integrity using 12 % SDS-PAGE gel electrophoresis. The CCM was then stored at −80 °C for further use.

### 2.4 Fabrication of CCM-coated ELP-based nanomicelles

To prepare the CCM solution, the MCF-7 cell membrane deposits were dissolved in PBS to a final concentration of 0.3 mg/mL. The NBD-labeled CCM (referred to as CCM-NBD) was prepared by dissolving 20 μg DSPE-NBD and 0.3 mg CCM in 1 mL PBS[28]. The CCM and CCM-NBD were sequentially extruded through both 1 μm and 400 nm polycarbonate membrane. For the IR780-loaded CCM (referred to as CCM/IR780), 100 μM IR780 was added to the CCM solution and extruded through both 1 μm and 400 nm polycarbonate membrane. The mixture was then subjected to ultrafiltration centrifugation at 5000 rpm, 4 °C for 30 min to obtain CCM/IR780. Subsequently, 500 μL of ELP-based micelles (ELP, ELP-Cy5, ELP-ICG, or ELP/Rapa, with a polypeptide concentration of 0.2 mg/mL, in PBS containing 0.5 % L-Arg) were separately mixed with 500 μL of freshly prepared cell membrane solutions (CCM, CCM-NBD, or CCM/IR780) in preassigned pairs. Each mixture was extruded through a 400 nm polycarbonate porous membrane for 10 cycles to form the CCM-coated ELP-based nanomicelles, including CCM@Arg/ELP, CCM@Arg/ELP-Cy5, CCM@Arg/ELP-ICG, CCM@Arg/ELP/Rapa, CCM-NBD@Arg/ELP-Cy5, and CCM/IR780@Arg/ELP/Rapa.

### 2.5 Characterization of nanomicelles

Hydrodynamic diameters of the nanomicelles including CCM@Arg/ELP, ELP, and non-cross-linked ELP were measured by dynamic light scattering (DLS) after incubation at 4 °C for 0, 6, 12, 24, and 48 h. The morphology of the nanomicelles (CCM@Arg/ELP and ELP) was examined using cryo-transmission electron microscopy (cryo-TEM). Their surface charge was assessed by measuring the zeta potentials. The CCM (in PBS containing 0.25 % L-Arg) was also characterized as described above as a control.

### 2.6 Analysis of drug encapsulation and responsive release capacity

The hydrophobic compound loading capacity of the nanomicelles was assessed with the help of 1,8-ANS. 200 μL of 25 μM non-cross-linked ELP/1,8-ANS at a polypeptide concentration of 0.2 mg/mL was prepared and followed by fluorescence measurement (λ_excitation_ = 370 nm) at 37 °C and 4 °C. Subsequently, the Rapa encapsulation efficiency of the CCM/IR780@Arg/ELP/Rapa and CCM@Arg/ELP/Rapa was analyzed with a high-performance liquid chromatography (HPLC) system. In brief, 100 μL of the Rapa-loaded nanomicelles was dissolved with 100 μL of acetonitrile and incubated at RT for 10 min to disrupt their structures. 100 μL of the sample was loaded onto the HPLC system, and the Rapa elution was monitored by absorption at 280 nm. The IR780 encapsulation efficiency of the CCM/IR780@Arg/ELP/Rapa was determined using UV spectrophotometry by recording the absorbance at 780 nm. The drug encapsulation rates were calculated according to the formula below:

$$\text{Encapsulation}\;\text{Efficiency}\;\left(\%\right)=\left(W_{e}/W_{i}\right)\times 100\%$$

where *W*_*e*_ was the total amount of encapsulated drugs and *W*_*i*_ was the initial amount of the drugs (i.e., total amount before encapsulation).

The Glutathione (GSH)-responsive Rapa release from the ELP/Rapa micelles was studied by dialysis and then quantified using HPLC. Briefly, 2.5 mL of ELP/Rapa with a polypeptide concentration of 0.2 mg/mL was diluted with 2.5 mL release buffer (1 mM PBS containing 20 mM GSH) and then placed into a rinsed dialysis tubing cellulose membrane with a 10 kDa molecular weight cutoff. The dialysis tube was sealed, placed into a 500 mL glass vial bottle containing 300 mL of the release buffer, and incubated at 25 °C, 200 rpm. The release buffer was replaced every 2 h to maintain sink conditions. The release buffer was collected and diluted with acetonitrile (v/v = 1:1) to dissolve the released Rapa. Aliquots of 100 μL were introduced into the HPLC system, and the Rapa elution was monitored by absorption at 280 nm. The release study conducted in the release buffer without GSH served as a control.

### 2.7 Study of internalization mechanism

MCF-7 cells were seeded in confocal dishes at a density of 1 × 10^4^ cells/mL and cultured in DMEM plus 10 % FBS (v/v) at 37 °C and 5 % CO_2_ for 48 h. 160 μL of each freshly prepared nanomicelle (CCM-NBD@Arg/ELP-Cy5, CCM@Arg/ELP-Cy5, and ELP-Cy5) was mixed with 40 μL 5 × DMEM prior to 2 h cell incubation. The cells were then washed three times with PBS, fixed with polyformaldehyde (4 %), stained with DAPI, and imaged using a laser scanning confocal microscope to evaluate the nanomicelle internalization. Additionally, to evaluate the targeted delivery capacity, mouse fibroblast L929 and breast cancer 4T1 cells were separately incubated with both CCM@Arg/ELP-Cy5 and ELP-Cy5 nanomicelles for 2 h before staining and imaging.

### 2.8 In vitro detection of ROS, NO, and mitochondrial activity

MCF-7 cells were seeded in a confocal dish at a density of 1 × 10^4^ cells/mL and cultured for 48 h. The CCM/IR780@Arg/ELP/Rapa, CCM@Arg/ELP/Rapa, and ELP/Rapa were separately added into the culture medium to a final equivalent Rapa concentration of 6.5 μM. The cells were cultured for an additional 2 h at 37 °C and 5 % CO_2_, rinsed with PBS, and incubated with 10 μM DCFH-DA or DAF-FM DA for 30 min in the dark. After 808 nm laser irradiation (1 W/cm^2^) for 5 min, the cells were fixed with 4 % paraformaldehyde, stained with DAPI, and imaged by a laser scanning confocal microscope for the evaluation of intracellular ROS or NO gas. PBS (containing 0.25 % L-Arg), 6.5 μM Rapa (in PBS containing 0.25 % L-Arg), and 0.4 μM IR780 (in PBS containing 0.25 % L-Arg) were employed as controls.

To evaluate the activity of mitochondria, MCF-7 cells were incubated with the CCM/IR780@Arg/ELP/Rapa, CCM@Arg/ELP/Rapa, or ELP/Rapa nanomicelles at an equivalent Rapa concentration of 6.5 μM for 2 h and then irradiated with an 808 nm laser (1 W/cm^2^) for 5 min. The cells were washed with PBS, cultured for an additional 46 h, stained with JC-1 assay kit according to the manufacturer's protocol, and imaged by a confocal microscope. PBS (containing 0.25 % L-Arg), 6.5 μM Rapa (in PBS containing 0.25 % L-Arg), and 0.4 μM IR780 (in PBS containing 0.25 % L-Arg) were employed as controls.

### 2.9 In vitro cytotoxicity assay

The antitumor effects of the CCM-coated ELP-based nanomicelles were evaluated in MCF-7 cells using LIVE/DEAD cell viability and CCK-8 assays. Cells were seeded in a 96-well plate at a density of 5000 cells/well and cultured in DMEM plus 10 % FBS (v/v) at 37 °C and 5 % CO_2_ for 48 h. Subsequently, the culture medium was replaced by the medium containing the CCM/IR780@Arg/ELP/Rapa, CCM@Arg/ELP/Rapa, or ELP/Rapa with an equivalent Rapa concentration of 6.5 μM. The cells were treated with nanomicelles for 2 h, gently rinsed with PBS for three times, irradiated with 808 nm laser (1 W/cm^2^) light for 5 min, and cultured in DMEM plus 10 % FBS (v/v) at 37 °C and 5 % CO_2_ for an additional 46 h. For the LIVE/DEAD cell viability assay, the cells were washed with PBS, stained with 2 μM of Calcein-AM/propidium iodide, and fixed with polyformaldehyde (4 %). The stained cells were imaged using an inverted fluorescence microscope with excitation/emission at 495 nm/515 nm and 493 nm/635 nm for the live and dead cells, respectively. The CCK-8 assay was conducted according to the manufacturer's protocol. A Plate Reader was employed to measure the absorbance at 450 nm of the cells, and the cell growth was calculated according to the formula below:

$$\text{Cell}\;\text{Viability}\;\left(\%\right)=\left({Abs}_{sample}/{Abs}_{control}\right)\times 100\%$$

where Abs_*sample*_ and Abs_*control*_ represent the absorbance at 450 nm of samples and controls. Meanwhile, PBS (containing 0.25 % L-Arg), Rapa (6.5 μM in PBS containing 0.25 % L-Arg), and IR780 (0.4 μM in PBS containing 0.25 % L-Arg) were used as controls.

### 2.10 Mouse tumor model

Female BALB/c nude mice (6–8 weeks, 18–20 g) were used to establish a unilateral tumor model. MCF-7 cells were collected, suspended in 200 μL PBS at a density of 1 × 10^7^ cells/mL, and then injected into the left second mammary fat pad of the mice. The in vivo experiments were conducted when the volume of the tumor tissues reached ∼100 mm^3^. All mice were raised in the Laboratory Animal Center of the First Hospital of Jilin University and maintained in a specific pathogen-free environment. All animal studies were performed following the guidelines for Experimental Animals of the First Hospital of Jilin University and approved by the Animal Experiment Ethics Committee (No. 11006).

### 2.11 In vivo evaluation of biodistribution

200 μL of the CCM@Arg/ELP-ICG and ELP-ICG nanomicelles at an equivalent ICG concentration of 50 μM were separately injected into the nude mice bearing the unilateral tumor through the tail vein. 50 μM ICG in 200 μL PBS containing 10 % L-Arg was utilized as a control. NIRvana® 640 SWIR camera was used to obtain the fluorescence images at 0.5, 2, 4, 6, 8, 10, 12, and 24 h post-injection. Subsequently, the mice were euthanized at 24 h post-injection. The tumor tissues and major organs (the heart, liver, spleen, lungs, and kidneys) were harvested, rinsed with PBS, and then imaged by the NIRvana® 640 SWIR camera. The average fluorescence was calculated with the help of Image J.

### 2.12 In vivo evaluation of antitumor effect

To evaluate the in vivo antitumor effect, unilateral tumor-bearing mice were subjected to intravenous injections via the tail vein on day 0, 2, 4, and 6. For each injection, 200 μL of nanomicelles (CCM/IR780@Arg/ELP/Rapa, CCM@Arg/ELP/Rapa, and ELP/Rapa) was administered at 13 μM rapamycin equivalent. On day 0, the tumor area was irradiated with 808 nm laser light (1 W/cm^2^) for 8 min at 6 h post-injection. The tumor volume and body weight of the mice were recorded daily. On day 14, all mice were sacrificed. The tumor tissue was collected, weighed, photographed, fixed in 4 % paraformaldehyde, and cut into 5 μm-thick slices. Tissue sections were subjected to hematoxylin and eosin (H&E) staining, terminal deoxynucleotidyl transferase dUTP nick-end labeling (TUNEL), and Ki-67 immunostaining. Meanwhile, Rapa (13 μM in PBS containing 1 % L-Arg), IR780 (15 μM in PBS containing 1 % L-Arg), and PBS (containing 1 % L-Arg) were used as controls.

### 2.13 Statistical analysis

All measurements were performed in triplicate, and all data were presented as mean ± standard deviation. Student's t-test using GraphPad Prism software was used to determine statistical significance. The significant difference of data was expressed as: *p < 0.05, **p < 0.01, ***p < 0.001, ****p < 0.0001.

## 3 Results and discussion

### 3.1 Formation and characterization of ELP-based micelles

**Fig. 1:**
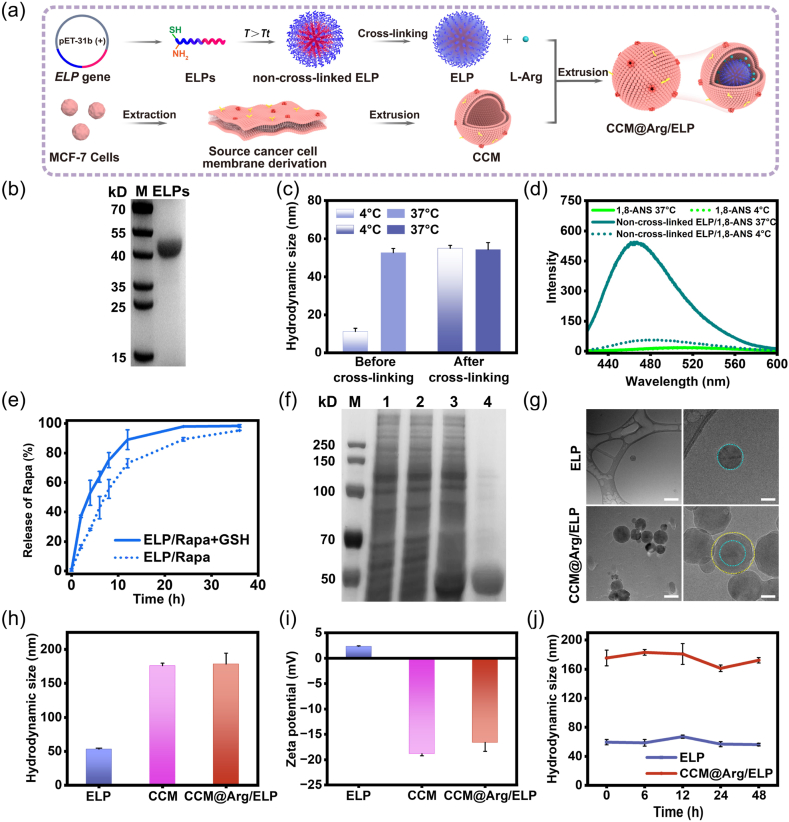
(a) Schematic illustration of the preparation of the CCM@Arg/ELP nanomicelles. (b) SDS-PAGE analysis of the diblock ELPs. (c) The hydrodynamic diameters of the ELP micelles with (right) or without (left) cross-linking treatment in PBS at 4 °C and 37 °C. (d) Fluorescence intensity of the 1,8-ANS molecules in the absence and presence of non-cross-linked ELP micelles at 4 °C and 37 °C, respectively. (e) Drug release profiles of the ELP/Rapa micelles in the presence or absence of GSH. (f) SDS-PAGE analysis of the CCM@Arg/ELP nanomicelles (lane 1: source cancer cell membrane derivation; lane 2: CCM; lane 3: CCM@Arg/ELP nanomicelles; lane 4: diblock ELPs). (g) Cryo-TEM images of the ELP and CCM@Arg/ELP nanomicelles (Scale bar is 200 nm (left) and 50 nm (right), respectively. The blue and yellow dashed lines outlined the inner ELP micelle and the outer CCM membrane, respectively). (h) The hydrodynamic diameter and (i) zeta potential of the ELP, CCM, and CCM@Arg/ELP. (j) The stability of the ELP and CCM@Arg/ELP nanomicelles incubated in PBS within 48 h at 4 °C (The replicate number is 3 in Fig. 1c, e,1h, 1i, and 1j). (For interpretation of the references to colour in this figure legend, the reader is referred to the Web version of this article.)

The diblock ELP was genetically engineered to contain a hydrophobic N-terminal (48 repeats of VPGIG) and a hydrophilic C-terminal (48 repeats of VPGSG followed by a GGKKGGKKGGCC sequence), as shown in Table S1. At temperatures above *T*_t_ (20 °C), this strategy enables ELPs to self-assemble into nanomicelles with the hydrophobic domains in the core and the hydrophilic domains displayed on the corona [17]. The hydrophobic chemotherapeutics can be encapsulated into the core of the ELP micelles. Additionally, the cysteines at the end of the hydrophilic domains allow corona cross-linking, enhancing stability during in vivo delivery and facilitating GSH-responsive release of the payloads within the tumor cells (Fig. 1a).

The ELPs were overexpressed in *E. coli* and purified by ITC. The result of SDS-PAGE shows that the band corresponding to the ELPs was around 40 kDa, confirming successful purification (Fig. S1 and 1b). The self-assembly of the ELPs into micelles in response to temperature change was evaluated by measuring the hydrodynamic diameters. As shown in Fig. 1c (left panel), the diameter was close to 10 nm at 4 °C, indicating the monomeric state of the ELPs. Upon increasing the temperature to 37 °C, the diameter of ELPs rose sharply to 54 nm with a PDI of 0.037, indicating micelle formation (the ELP micelles prepared via the thin-film hydration method exhibited a similar diameter, see Fig. S2). Given that most micelles tend to disassemble in biological fluids and prematurely release the payloads, our ELP micelles were designed to allow cystine-derived corona cross-linking for stability improvement [29]. After cross-linking, the ELP micelles could maintain their diameters (∼54 nm with a PDI of 0.073) at both 4 °C and 37 °C, indicating structural stability (Fig. 1c, right panel). These results collectively demonstrate that our diblock ELPs can self-assemble into micelles at physiological temperature and achieve structure stabilization through cystine-derived corona cross-linking.

### 3.2 Hydrophobic compound encapsulation and GSH-responsive release of ELP-based micelles

Considering that most micelles can stabilize the hydrophobic compounds in the cores and thus prolong their circulation in the bloodstream, the hydrophobic compounds encapsulation capacity of our ELP micelles was evaluated [27]. The hydrophobic environment-sensitive dye 1,8-ANS was employed as the model hydrophobic drug. The PBS solution containing 25 μM of the dye and 0.2 mg/mL of the ELPs was prepared and then individually measured for fluorescence intensity at 4 °C and 37 °C. As shown in Fig. 1d, almost no fluorescence emission signals were detected in all groups at 4 °C. Notably, when the temperature increased to 37 °C, a dramatic increase in fluorescence emission signals could be detected with the sample containing both ELPs and 1,8-ANS. The elevated fluorescence intensity is attributed to the encapsulation of 1,8-ANS molecules into the hydrophobic core of the ELP micelles that self-assembled at 37 °C. This observation demonstrates that our ELP micelles can encapsulate hydrophobic compounds efficiently, which is in good correlation with the previous reports [15].

Elevated activation of the mammalian target of the rapamycin (mTOR) signaling pathway, which has been proven to endow cancer cells with a growth advantage, has been observed in various human cancers including breast cancer [[30], [31], [32]]. Consequently, rapamycin (Rapa), a specific inhibitor of mTOR, has become one of the most commonly used anticancer drugs for breast cancer treatment in the clinic [33,34]. However, the poor water solubility of Rapa severely impacts its anticancer efficacy during clinical application [35]. The ELP micelles were therefore utilized to encapsulate Rapa based on hydrophobic interactions. The Rapa-loaded ELP (ELP/Rapa) micelles were prepared using the thin film hydration method followed by cross-linking treatment and then characterized for the encapsulation rates by HPLC. The ELP/Rapa micelles exhibited an encapsulation rate of 30 %, which is in accordance with the previously reported hydrophobic compound loading capacity of the ELP-based micelles. Given that an ideal drug carrier should not only retain the payloads during in vivo delivery but also release them efficiently in target cells, the GSH-responsive release property of the ELP micelles was evaluated. As shown in Fig. 1e, a significantly faster release rate can be achieved with the ELP/Rapa micelles in the presence of GSH within 12 h. Notably, the release rate of the ELP/Rapa with GSH group was 36 % and 53 % at 2 h and 4 h, respectively, which is two times as high as that of the ELP/Rapa without GSH group at the same time interval. Furthermore, the release advantage persisted at 6 h, 8 h, and 12 h, with the GSH group exhibiting 1.5-, 1.4-, and 1.2-fold higher release rates, respectively. These results demonstrate that our ELP micelles can efficiently encapsulate hydrophobic chemotherapeutics in the core based on hydrophobic interactions and then expeditiously release the payloads through the GSH-responsive cleavage of the disulfide bonds within the corona. Notably, the CCM coating acts as a robust barrier that effectively limits the diffusion and premature release of Rapa, thereby enhancing its in vivo delivery efficiency (Fig. S3).

### 3.3 Fabrication and characterization of CCM-coated ELP-based nanomicelles

Most drug delivery systems utilize the endocytic pathway for cellular entry, a process in which the plasma membrane engulfs the drug carrier to form a vesicle. Following endocytosis, drug carriers are transported to early endosomes, which subsequently mature into late endosomes and ultimately fuse with lysosomes. For efficient delivery, the payload must be released into the cytosol before late endosomes mature into lysosomes, where most exogenous materials are enzymatically degraded. However, escape from the endosomal-lysosomal pathway remains inefficient for most drug carriers. Typically, less than 2 % of the internalized cargo reaches the cytoplasm, making endolysosomal escape a major bottleneck in the field. In contrast, fusogenic cell membranes can be applied as carrier coatings, enabling drug vehicles to bypass the conventional endocytic pathway and achieve direct cytosolic delivery, thereby improving intracellular transport efficiency [[36], [37], [38], [39]]. To achieve this goal, the ELP micelles were coated with the membranes of MCF-7 cells, a breast cancer cell line possessing a homologous cell fusion character. MCF-7 cells have been reported to exhibit homologous targeting and binding capabilities both in vitro and in vivo [40]. Following such targeting, MCF-7 membrane-coated nanoparticles have been demonstrated to undergo homologous membrane fusion [21]. The cell membranes were obtained through freezing and thawing MCF-7 cells with the help of a membrane protein extraction kit. Fig. 1f shows that the obtained CCM possessed a protein profile similar to that of the MCF-7 cell membrane derivation. It, therefore, demonstrates that the as-prepared CCM retained most endogenous cell membrane proteins, which allow MCF-7 cells to recognize and fuse with homologous cells. The CCM, ELP micelles, and L-Arg molecules were mixed and the mixture was extruded through a 400 nm polycarbonate porous membrane for 10 cycles to fabricate the CCM@Arg/ELP nanomicelles. As shown in Fig. 1f, the results of SDS-PAGE revealed that the bands corresponding to the diblock ELPs and the membrane proteins of the CCM can be observed in the protein profile of the CCM@Arg/ELP nanomicelles. The Cryo-TEM imaging shows that both CCM@Arg/ELP and ELP nanomicelles displayed a spherical morphology (Fig. 1g). The diameter of the CCM@Arg/ELP sample was measured to be 176 nm with a PDI of 0.198, which is similar to that of the CCM control but 120 nm larger than that of the ELP micelles (Fig. 1h). Collectively, these observations demonstrate the successful coating of the ELP micelles with the CCM. Moreover, the average zeta potential of the CCM@Arg/ELP nanomicelles was intermediate between those of the CCM and ELP micelles (Fig. 1i), confirming the successful coating. The more positive average zeta potential of the CCM@Arg/ELP nanomicelles compared with the CCM is attributed to the encapsulation of the positively charged ELP micelles and L-Arg molecules. The loading efficiency of L-Arg was consistent across replicate experiments, as shown in Table S2. The stability of the CCM@Arg/ELP nanomicelles was then carefully characterized. Similar to the ELP micelles, the CCM@Arg/ELP nanomicelles can maintain their particle size well after incubation at 4 °C in PBS solution for 48 h, indicating excellent stability (Fig. 1j).

### 3.4 Evaluation of homotypic membrane fusion capacity of CCM-coated ELP-based nanomicelles

**Fig. 2:**
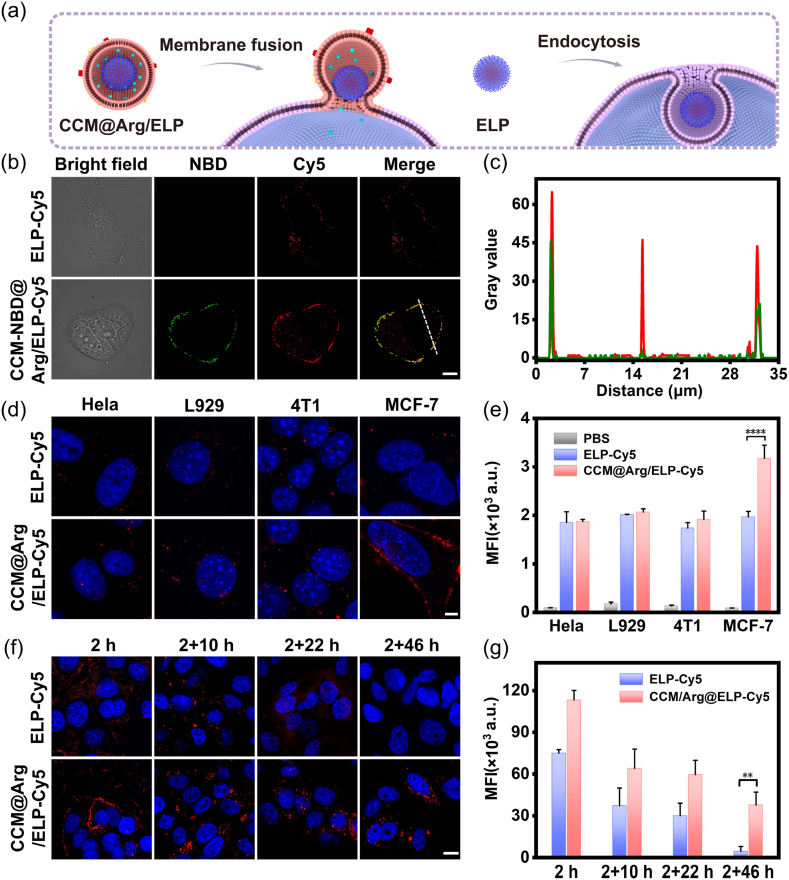
(a) Schematic diagram depicting membrane fusion and endocytosis pathways. (b) CLSM images depicting the homotypic membrane fusion between the nanomicelles (CCM-NBD@Arg/ELP-Cy5 or ELP-Cy5) and MCF-7 cells after 2 h incubation. (c) Gray value quantification in red and green channels along the white dashed line indicated in Fig. 2a using ImageJ software. (d) CLSM imaging and (e) FACS analysis of homotypic targeting capacity of the CCM@Arg/ELP-Cy5 and ELP-Cy5 nanomicelles towards HeLa, L929, 4T1, and MCF-7 cells after 2 h incubation. (f) CLSM images and (g) semi-quantitative fluorescence intensity analysis of MCF-7 cells treated with the CCM@Arg/ELP-Cy5 or ELP-Cy5 nanomicelles for 2 h, followed by nanomicelle-free incubation for 10 h, 22 h, or 46 h. Red, green, and blue fluorescence corresponded to ELP-Cy5, CCM-NBD, and DAPI-stained nucleus, respectively. Scale bar in Figs. b, d, and f is 10, 10, and 15 μm, respectively (The replicate number is 3 in Fig. 2e and g). (For interpretation of the references to colour in this figure legend, the reader is referred to the Web version of this article.)

To demonstrate that the CCM-coated ELP-based nanomicelles can fuse with the breast cancer cells followed by delivering the payloads into the cytoplasm (Fig. 2a, left panel), the CCM shell and the ELP core were labeled with NBD (green) and Cy5 (red), respectively. The labeled nanomicelles (CCM-NBD@Arg/ELP-Cy5 and ELP-Cy5) were then incubated with MCF-7 cells for 2 h, followed by imaging with CLSM and analyzing using ImageJ software. In the CCM-NBD@Arg/ELP-Cy5 group, the green fluorescent signals corresponding to the CCM-NBD coating were observed to spread along the cell membrane (Fig. 2b), demonstrating membrane fusion [41]. For the red fluorescence of the ELP-Cy5 cores, some of them colocalized well with the green fluorescence of the CCM-NBD coating, indicating that the ELP-Cy5 cores had not yet separated from the CCM, whilst the rest were found to diffuse into the cytoplasm (Fig. 2b and c) [42]. It indicates that the CCM-NBD@Arg/ELP-Cy5 nanomicelles delivered the ELP-Cy5 cores into MCF-7 cells through membrane fusion. In contrast, in the ELP-Cy5 (the micelles without CCM coating) group, almost all the red fluorescent signals were observed to be located within the cells (Fig. 2b), suggesting endocytosis-based uptake pathways (Fig. 2a, right panel) [43]. The lack of lysosomal colocalization (by LysoTracker Green staining, Fig. S4) and the insensitivity to endocytic inhibitors (Fig. S5) collectively demonstrate that the CCM-coated nanomicelles evade the classical endolysosomal pathway. Membrane fusion capacity of the CCM-coated nanomicelles was also confirmed in experiments pairing CCM-NBD with MCF-7 cells and membrane fusogenic liposomes with MCF-7 cells. The CCM-coated nanomicelles possess comparable membrane fusion capacity with conventional membrane-fusogenic liposomes (Fig. S6). These independent lines of evidence strongly support the conclusion that the CCM coating enables direct cytosolic delivery through membrane fusion with MCF-7 cells.

To verify the homotypic targeting capacity of the CCM-coated ELP-based nanomicelles, HeLa, L929, and 4T1 cells were also incubated with the labeled nanomicelles (CCM@Arg/ELP-Cy5 and ELP-Cy5) as described above [44]. The fluorescence images and FACS data are shown in Fig. 2d and e, respectively. In the CCM@Arg/ELP-Cy5 group, significantly fewer fluorescent signals were observed in HeLa, L929, and 4T1 cells compared to MCF-7 cells, demonstrating less internalization. In contrast, after incubation with the ELP-Cy5 micelles, no obvious difference in the fluorescent intensity can be detected in HeLa, L929, 4T1, and MCF-7 cells, indicating similar internalization efficiency. Taken together, it can be concluded that the CCM coating allows the CCM-coated ELP-based nanomicelles to self-recognize homologous cells, which can be utilized to facilitate targeted delivery for breast cancer treatment [45].

It has been reported that the membrane fusion strategy enables the nanoparticles to bypass the endocytosis pathway, thereby improving intracellular trafficking [[46], [47], [48], [49], [50]]. To demonstrate this, MCF-7 cells were incubated with the CCM@Arg/ELP-Cy5 and ELP-Cy5 nanomicelles for 0.5, 1, 2, and 4 h, washed with PBS to remove non-internalized nanomicelles, stained with Hoechst and imaged by confocal laser scanning microscopy. As shown in Fig. S7, the Cy5 fluorescence intensity (red) of the CCM@Arg/ELP-Cy5 group was markedly higher than that of the ELP-Cy5 group at all time points. This result demonstrates the superior performance of the membrane fusion-based delivery strategy over the endocytosis-mediated pathway. To further study the intracellular trafficking, the cells incubated with the nanomicelles for 2 h were washed with PBS, and then cultured for an additional 10 h, 22 h, or 46 h. The cells were subsequently subjected to CLSM imaging at each time point, and the images were analyzed using ImageJ software. Following the removal of non-endocytosed nanomicelles, intracellular Cy5 fluorescence intensity decreased over time in cells treated with either CCM@Arg/ELP-Cy5 or ELP-Cy5 nanomicelles (Fig. 2f and g), attributable to nanomicelle disassembly, degradation post-internalization, and further metabolism [51,52]. Notably, the CCM@Arg/ELP-Cy5 group exhibited significantly higher red fluorescent intensity than the ELP-Cy5 group at all time points (Fig. 2f and g), demonstrating enhanced intracellular accumulation [53]. This is because the CCM@Arg/ELP-Cy5 nanomicelles directly deliver payloads into the cytoplasm, bypassing the endosomal-lysosomal system, which degrades most entrapped nanoparticles [54]. These observations demonstrate that our CCM-coated ELP-based nanomicelles can efficiently enter breast cancer cells and achieve robust intracellular accumulation, benefiting from the homotypic membrane fusion mechanism of the CCM coating.

### 3.5 Evaluation of membrane damage induced by anchored photosensitizer

**Fig. 3:**
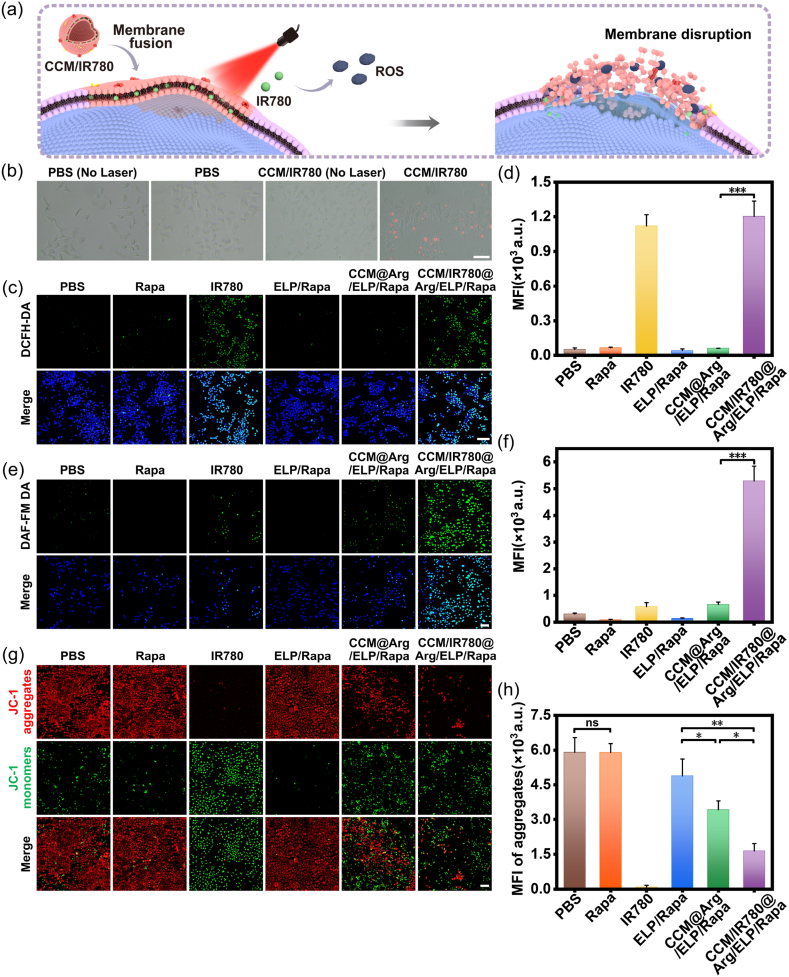
(a) Schematic illustration of CCM/IR780-mediated photosensitizer anchoring to target cell membranes, followed by membrane disruption upon NIR irradiation. (b) Fluorescence microscopy images of plasma membrane integrity in MCF-7 cells following the CCM/IR780@Arg/ELP/Rapa nanomicelle delivery with or without NIR irradiation using PI staining (red: PI). (c) CLSM images and (d) semi-quantitative fluorescence intensity analysis of ROS generation in MCF-7 cells incubated with nanomicelles or free compounds for 2 h using DCFH-DA probe (green: DCF). (e) CLSM images and (f) semi-quantitative fluorescence intensity analysis of NO generation in MCF-7 cells after incubation with nanomicelles or free compounds for 2 h using DAF-FM DA probe (green: DAF-FM). (g) CLSM images and (h) semi-quantitative fluorescence intensity analysis of mitochondrial membrane potential in MCF-7 cells treated with nanomicelles or free compounds for 2 h followed by culture in agent-free medium for 46 h (red: J-aggregate; green: J-monomer). Scale bars: b, e, g = 100 μm; c = 150 μm (The replicate number is 3 in Fig. 3d, f, and 3h). (For interpretation of the references to colour in this figure legend, the reader is referred to the Web version of this article.)

As reported, the cell membrane-based materials possessing membrane fusion capability enable photosensitizer anchoring onto the target cell membrane and induce membrane damage during PDT (Fig. 3a) [55,56]. To validate this feature, IR780, a near-infrared fluorescent dye that can be applied as a photosensitizer [57], was encapsulated within the CCM (with an encapsulation rate of 19.2 %, see Fig. S8) and incubated with MCF-7 cells for 2 h. The confocal scanning imaging shows that the CCM/IR780 group shows significant colocalization (yellow signals) of the IR780 signal with the Dil-stained cell membrane, confirming successful anchoring of IR780 to the cell membrane (Fig. S9). Following the removal of non-internalized nanomicelles and an additional 4-h culture period, the IR780 signal remained predominantly localized at the cell membrane (Fig. S10). Following NIR irradiation after the initial 2 h incubation to initiate PDT, the integrity of the cell membrane was evaluated using propidium iodide (PI), a red fluorescence DNA intercalating reagent that permeates compromised membranes. Fig. 3b shows that distinct PI fluorescence (red) was observed exclusively in the cells treated with the CCM/IR780 nanomicelles under NIR irradiation, indicating PDT-induced tumor cell death [58]. Based on these observations, it suggests that our CCM-coated nanoparticles can anchor photosensitizers to breast cancer cell membranes via membrane fusion, which may cause cell membrane damage and thereby potentiate PDT-induced tumor cell death [59].

Studies have shown that photosensitizers can convert endogenous oxygen into cytotoxic reactive oxygen species (ROS) during PDT [22]. Consequently, we hypothesized that the plasma membrane damage observed in Fig. 3b was resulted from ROS generated by the cell membrane-anchored IR780 upon NIR irradiation (Fig. 3a). To test this hypothesis, MCF-7 cells were incubated with the CCM/IR780@Arg/ELP/Rapa nanomicelles as described, and the intracellular ROS generation was assessed using the fluorescent ROS probe DCFH-DA. Control groups included the CCM@Arg/ELP/Rapa, ELP/Rapa, IR780, Rapa, and PBS. As shown in Fig. 3c, negligible green fluorescent signals were detected in the cells treated with CCM@Arg/ELP/Rapa, ELP/Rapa, Rapa, and PBS, indicating that limited intracellular ROS was generated in IR780-free conditions. In sharp contrast, both the CCM/IR780@Arg/ELP/Rapa and free IR780 groups exhibited intense green fluorescence, demonstrating substantial intracellular ROS production induced by NIR irradiation of IR780. The quantified mean fluorescence intensity corroborated these observations (Fig. 3d). Collectively, these findings demonstrate that the developed CCM-coated ELP-based nanomicelles effectively anchor photosensitizers to the membranes of breast cancer cell membranes and subsequently generate ROS during PDT to induce direct membrane damage. Although ROS can deplete intracellular GSH, their impact on GSH-mediated Rapa release from ELP micelles is likely limited due to the transient nature of ROS (lifetimes in the nanosecond to microsecond range) and the high basal GSH levels in cancer cells, which ensure sufficient reducing capacity remains to drive the sustained disulfide cleavage and Rapa release process [24,60].

### 3.6 In vitro evaluation of the generation of NO and mitochondrial activity

Beyond inducing oxidative stress, ROS generated during PDT can oxidize L-Arg to produce NO gas [61,62], which exerts tumoricidal effects [63,64]. To quantify NO production, MCF-7 cells incubated with the nanomicelles for 2 h were subjected to NIR irradiation and stained with DAF-FM DA fluorescent probe. The CLSM images and semi-quantitative analysis revealed that the strongest green fluorescence signals corresponding to NO gas were observed in the CCM/IR780@Arg/ELP/Rapa group, demonstrating efficient NO generation (Fig. 3e and f). In contrast, NO fluorescence intensity decreased dramatically (more than 87.5 %) within the cells treated with the CCM@Arg/ELP/Rapa nanomicelles or free IR780, indicating limited intracellular NO production in the absence of photosensitizers or L-Arg encapsulated nanomicelles, respectively. The limited NO generation in the CCM@Arg/ELP/Rapa group likely stems from endogenous enzymatic NO synthesis utilizing the cytoplasmically released L-Arg, whereas in the free IR780 group, it arises from ROS-mediated oxidation of intracellular L-Arg. Furthermore, negligible green fluorescence signals were observed in the ELP/Rapa, Rapa, and PBS groups, which lacked both photosensitizers and L-Arg encapsulated nanomicelles. These results collectively demonstrate that the CCM/IR780@Arg/ELP/Rapa nanomicelles enable intracellular NO generation via L-Arg oxidation by ROS generated during PDT, following photosensitizer anchoring to the cell membrane and cytoplasmic release of L-Arg [65].

Both ROS and NO generated during PDT have been reported to induce mitochondrial dysfunction, leading to irreversible cancer cell apoptosis [23,63,66,67]. To assess mitochondrial activity in our system, the mitochondrial membrane potential of MCF-7 cells was assessed using JC-1 staining after the treatment with nanomicelles in the presence of NIR light. JC-1 forms red fluorescence aggregates in healthy mitochondria, but converts to green fluorescent monomers upon membrane potential depolarization. As shown in Fig. 3g, the dual ROS/NO-generating nanomicelles (CCM/IR780@Arg/ELP/Rapa) resulted in significantly fewer red fluorescence aggregates in cells than the negligible-NO-generating nanomicelles (CCM@Arg/ELP/Rapa), non-CCM-coated nanomicelles (ELP/Rapa), and free compounds (Rapa and PBS). The semi-quantitative analysis in Fig. 3h confirmed the reduced functional mitochondria in the CCM/IR780@Arg/ELP/Rapa-treated cells. Concurrently, the CCM/IR780@Arg/ELP/Rapa group exhibited substantially enhanced green fluorescence signals (Fig. 3g), indicating ROS/NO-induced mitochondrial membrane potential depolarization [64,68]. These results demonstrate that despite not targeting mitochondria specifically (Fig. S11), the CCM/IR780@Arg/ELP/Rapa nanomicelles can induce severe mitochondrial damage in breast cancer cells through the generation of ROS and NO under NIR irradiation. Free IR780 also significantly impaired the mitochondria function due to its cell permeable nature [69], which facilitated ROS-mediated oxidation of intracellular L-Arg during PDT (Fig. 3g and h).

### 3.7 Antitumor activity in vitro

**Fig. 4:**
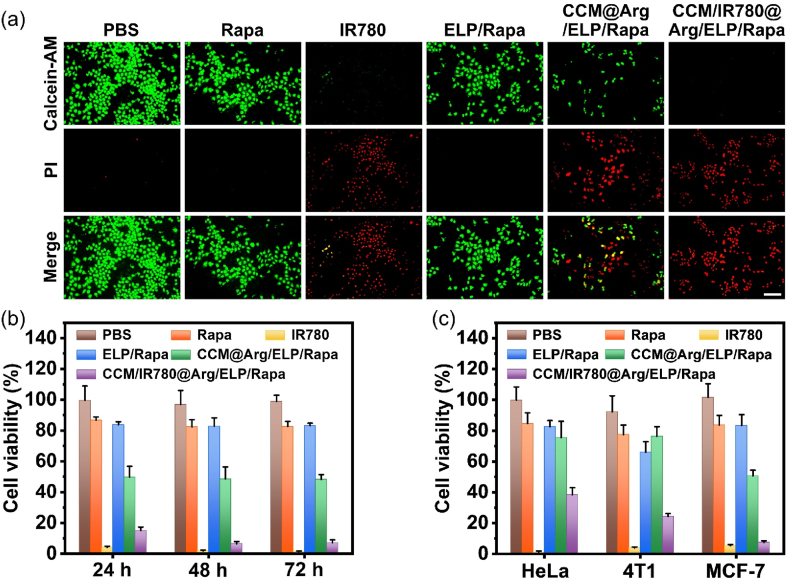
(a) LIVE/DEAD staining of MCF-7 cells incubated with nanomicelles or free compounds (scale bar: 100 μm). (b) Time-dependent viability of MCF-7 cells after incubation with nanomicelles or free compounds. (c) Viability of HeLa, 4T1, and MCF-7 cells following incubation with nanomicelles or free compounds. All treatments consisted of 2 h incubation with nanomicelles or free compound under NIR irradiation, followed by (a) 46 h, (b) 22/46/70 h, or (c) 46 h culture in agent-free medium (The replicate number is 6 in Fig. 4b and c).

To achieve the synergistic effect of membrane fusion, PDT, gas therapy, and chemotherapy, the MCF-7 cells were treated with nanomicelles for 2 h. After nanomicelle removal and NIR irradiation, the cells were maintained for another 22, 46, or 70 h. As shown in Fig. 4a, LIVE/DEAD staining at 48 h revealed substantially enhanced red fluorescence (indicating dead cells) in the CCM/IR780@Arg/ELP/Rapa group compared to all controls (CCM@Arg/ELP/Rapa, ELP/Rapa, Rapa, and PBS), demonstrating potent antitumor efficacy. CCK-8 assay further confirmed this marked antitumor efficacy, with the CCM/IR780@Arg/ELP/Rapa nanomicelles resulting in cell viability of 15.1 % at 24 h, 6.5 % at 48 h, and 7.1 % at 72 h (Fig. 4b). These values were significantly lower than those of the CCM@Arg/ELP/Rapa (49.7 %, 48.5 %, and 48.2 %), ELP/Rapa (83.8 %, 82.8 %, and 83.1 %), and Rapa (86.6 %, 82.4 %, and 82.6 %) at respective time points. Additionally, the ELP and CCM components maintained nearly 100 % cell viability across all culture durations, revealing excellent biocompatibility (Fig. S12). Collectively, the potent antitumor efficacy of the CCM/IR780@Arg/ELP/Rapa nanomicelles arises from synergistic integration of CCM-mediated membrane fusion, IR780-triggered PDT, L-Arg-derived gas therapy, and Rapa-induced cytotoxicity. The antitumor effect of free IR780 may be attributed to its non-specific adsorptive endocytosis or diffusion [70], which induces PDT within breast cancer cells upon NIR irradiation (Fig. 4a and b).

The targeted cytotoxicity of the CCM-coated ELP-based nanomicelles toward breast cancer cells was further evaluated using MCF-7, HeLa, and 4T1 cells. The cells were incubated with nanomicelles for 2 h, followed by NIR irradiation [67,71,72]. After nanomicelle removal, the cells were cultured for an additional 46 h prior to viability assessment. As shown in Fig. 4c, the CCM/IR780@Arg/ELP/Rapa treatment resulted in cell viability of 38.4 % in HeLa cells and 24.2 % in 4T1 cells, but only 7.3 % in MCF-7 cells, indicating substantial targeting specificity attributable to homotypic membrane fusion [37]. The other CCM-coated nanomicelle (CCM@Arg/ELP/Rapa) also induced significantly lower viability in MCF-7 cells (50.6 %) compared to HeLa (75.5 %) and 4T1 cells (76.3 %), confirming CCM coating-dependent targeting. In sharp contrast, treatment with the ELP/Rapa, IR780, or Rapa alone, showed similar cytotoxicity across all three cell lines. These results validate the selective cytotoxicity of the CCM-coated ELP-based nanomicelles toward breast cancer cells as anticipated [73]. Taken together, the superior antitumor capacity of our CCM/IR780@Arg/ELP/Rapa nanomicelles originates from the following factors: i) CCM mediated homotypic targeting and membrane fusion, anchoring IR780 to cancer cell membranes while enabling direct cytoplasmic delivery of L-Arg and ELP/Rapa payloads; ii) PDT triggered by membrane-anchored IR780 upon NIR irradiation, causing direct membrane damage; iii) L-Arg conversion to NO during PDT, inducing mitochondrial dysfunction; iv) GSH-triggered cleavage of corona disulfide bonds in ELP/Rapa micelles, enhancing Rapa release.

### 3.8 In vivo biodistribution of CCM-coated ELP-based nanomicelles

**Fig. 5:**
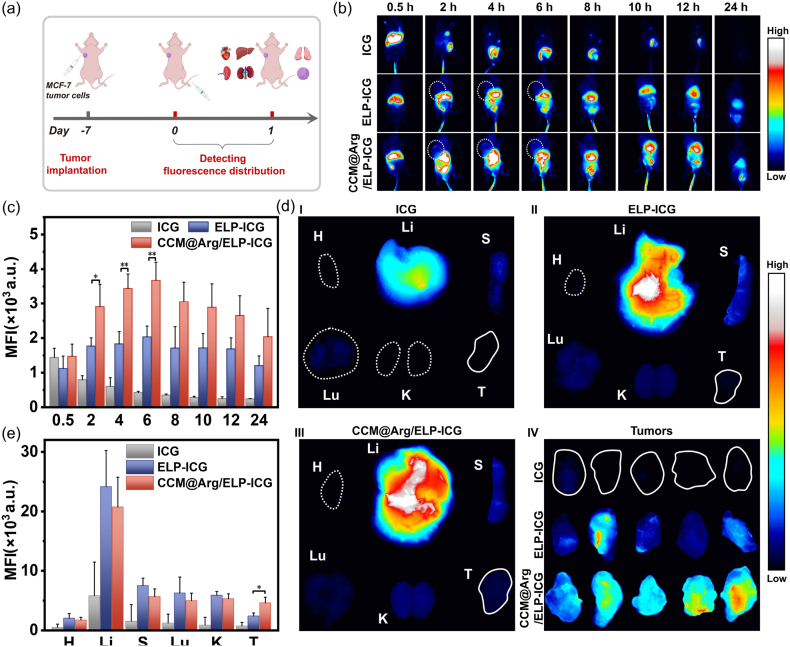
(a) Scheme of in vivo biodistribution evaluation. (b) In vivo fluorescence images and (c) the semi-quantitative intensity analysis of tumor-bearing mice at various time points post-administration of the CCM@Arg/ELP-ICG, ELP-ICG, or free ICG (n = 3). (d) Ex vivo fluorescence images of tumors and major organs acquired 24 h after administration of free ICG (I), ELP-ICG (II), or CCM@Arg/ELP-ICG (III). IV: Corresponding in vitro fluorescence images of tumors from each group at 24 h (n = 5). (e) The semi-quantitative intensity analysis of tumors and major organs acquired 24 h after administration of free ICG, ELP-ICG, or CCM@Arg/ELP-ICG (n = 5) (H: Heart; Li: Liver; S: Spleen; Lu: Lung; K: Kidney; T: Tumor).

Encouraged by the robust in vitro antitumor efficacy of the CCM-coated ELP-based nanomicelles, we subsequently evaluated their tumor accumulation capacity. Due to commercial availability of NHS modified ICG molecules, the ICG conjugated nanomicelles (CCM@Arg/ELP-ICG and ELP-ICG) were prepared. The nude mice bearing the unilateral tumors were intravenously injected with the ICG conjugated nanomicelles (CCM@Arg/ELP-ICG and ELP-ICG) or free ICG, followed by in vivo fluorescence imaging upon NIR irradiation at 24 h (Fig. 5a). In vivo fluorescence images and the semi-quantitative intensity analysis are shown in Fig. 5b and c. At 0.5 h post-injection, all groups (CCM@Arg/ELP-ICG, ELP-ICG, and ICG) showed fluorescence signals within tumor sites, indicating tumor delivery via systemic circulation. In the CCM@Arg/ELP-ICG group, the fluorescence intensity at tumor sites progressively increased and reached the maximum at 6 h post-injection (Fig. 5b and c), demonstrating CCM-mediated active tumor targeting [74]. Notably, preferential tumor retention persisted even at 24 h in the CCM@Arg/ELP-ICG group. While the ELP-ICG micelles exhibited similar temporal accumulation kinetics in tumor sites due to passive EPR targeting [75,76], their tumor fluorescence intensity was significantly lower than the CCM@Arg/ELP-ICG nanomicelles (Fig. 5b and c). Conversely, free ICG showed rapid fluorescence signal decay over time, indicating poor tumor retention. Similarly, it has been reported that free IR780, L-Arg as well as Rapa, do not exhibit ideal tumor accumulation due to their rapid systemic clearance, non-specific distribution, and lack of active targeting.

To further investigate the in vivo biodistribution of the nanomicelles, the major organs and tumor tissues were collected 24 h post-injection for ex vivo imaging (Fig. 5a and d). As shown in Fig. 5d and e, the free ICG group showed negligible fluorescence signals in all organs and tumor tissues, attributed to rapid clearance [77]. Both CCM@Arg/ELP-ICG and ELP-ICG nanomicelles exhibited similar organ retention in the liver, lungs, kidneys, spleen, and heart, indicating comparable reticuloendothelial system (RES) clearance pathways [[78], [79], [80]]. Consistent with this, the free components L-Arg and Rapa are also known to accumulate primarily in the liver [[81], [82], [83], [84]]. Notably, the CCM@Arg/ELP-ICG nanomicelles resulted in stronger fluorescence in tumor tissues compared with the ELP-ICG nanomicelles (Fig. 5d and S13). The semi-quantitative analysis shown in Fig. 5e confirmed the enhanced tumor retention of the CCM@Arg/ELP-ICG nanomicelles. These results demonstrate efficient tumor-selective accumulation and prolonged tumor retention of the CCM-coated ELP-based nanomicelles, which are essential pharmacokinetic properties for anticancer efficacy [85].

### 3.9 In vivo antitumor performance

**Fig. 6:**
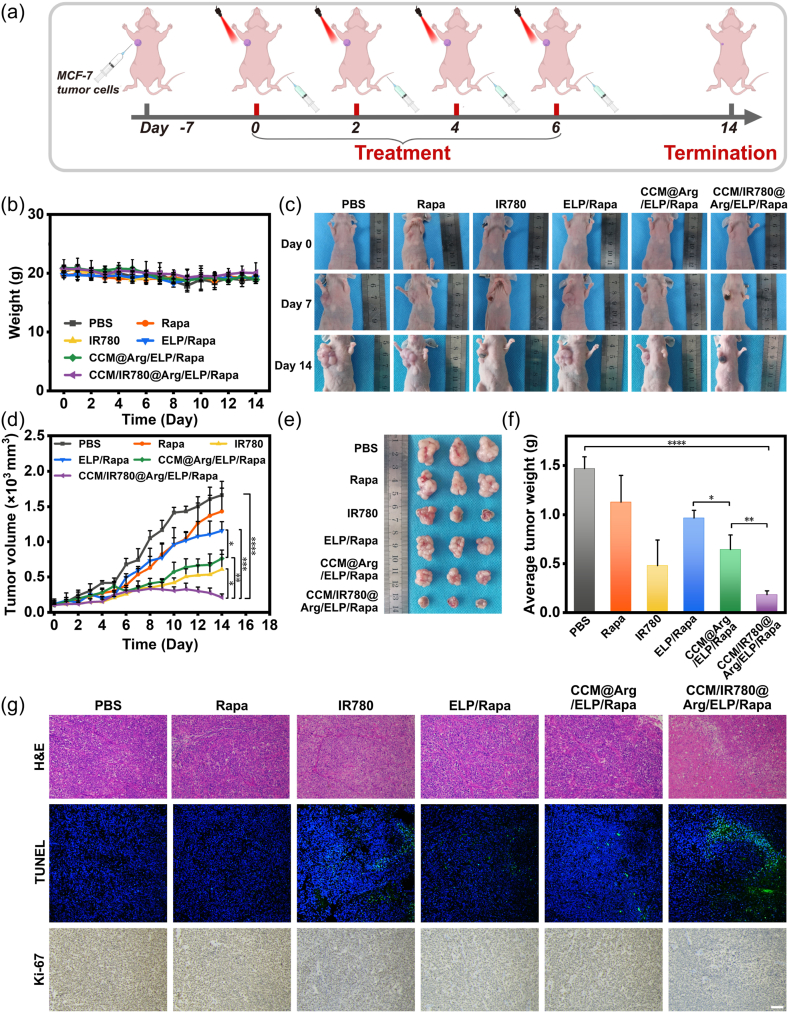
(a) Scheme of in vivo antitumor performance evaluation. (b) Body weight monitoring of tumor-bearing mice treated by nanomicelles or free compounds during treatment. (c) Representative photographs of tumor-bearing mice treated by nanomicelles or free compounds on day 0, 7, and 14. (d) Tumor volume progression of tumor-bearing mice following different treatments. (e) Representative photos of harvested tumors in each group on day 14. (f) The weight of the excised tumors of the mice following different treatments on day 14. (g) H&E staining, TUNEL assay, and Ki-67 immunohistochemistry of tumor tissues of the mice after 14-day treatment (Scale bar: 100 μm). Each group contained three independent replicates (n = 3).

To directly evaluate the antitumor efficacy of our CCM-coated ELP-based nanomicelles, the unilateral tumor-bearing mice were intravenously injected with 200 μL of CCM/IR780@Arg/ELP/Rapa, CCM@Arg/ELP/Rapa, ELP/Rapa, IR780, Rapa, or PBS on day 0, 2, 4, and 6, with all mice receiving NIR irradiation for 8 min at 6 h post-injection (Fig. 6a). The therapeutic effect of L-Arg depends critically on ROS generated during IR780-induced PDT. In the absence of a sufficient ROS, free L-Arg cannot be efficiently oxidized to NO, and thus does not exhibit gas therapy efficacy. Free L-Arg was not included as a control group. The body weight and tumor volume were monitored every other day. As shown in Fig. 6b, the mice treated with the nanomicelles exhibited negligible weight loss, comparable to the PBS control, indicating excellent biocompatibility [86]. H&E staining of major organs (heart, liver, spleen, lungs, and kidneys) further demonstrated the negligible toxicity of our nanomicelles (Fig. S14). After 14-day treatment, obvious tumor necrosis was observed in the CCM/IR780@Arg/ELP/Rapa group (Fig. 6c and S15). Notably, tumors in the CCM/IR780@Arg/ELP/Rapa group showed slight growth before day 8, followed by gradual shrinkage from day 9 onwards (Fig. 6c and d, and S15). In contrast, tumor volume increased progressively over the 14-day period in the mice treated with the CCM@Arg/ELP/Rapa, ELP/Rapa, IR780, Rapa, or PBS, demonstrating limited tumor suppression capacity (Fig. 6c and d). These results verify the potent in vivo antitumor activity of the CCM/IR780@Arg/ELP/Rapa nanomicelles. To further assess the antitumor effect, all mice were sacrificed on day 14. The tumor tissues and major organs were isolated and collected. Representative photographs of the isolated tumor tissues revealed the smallest tumor volume in the CCM/IR780@Arg/ELP/Rapa group compared to other treatments (Fig. 6e). The average tumor weight in the CCM/IR780@Arg/ELP/Rapa group was 0.18 g, representing an 87.7 % reduction compared to that of the PBS control (1.47 g) (Fig. 6f). In contrast, the average tumor weights for the CCM@Arg/ELP/Rapa, ELP/Rapa, IR780, and Rapa groups were 0.64 g, 0.97 g, 0.48 g, and 1.13 g, corresponding to a tumor weight inhibition rate of 56.5 %, 34.0 %, and 67.3 %, and 23.1 % respectively. These findings unequivocally demonstrate the superior antitumor performance of the CCM/IR780@Arg/ELP/Rapa nanomicelles.

Meanwhile, the H&E staining of the tumor tissue sections revealed the largest necrotic areas in the CCM/IR780@Arg/ELP/Rapa group compared to other groups. Moreover, TUNEL staining demonstrated the most severe DNA fragmentation (green fluorescence) in the tumors treated with the CCM/IR780@Arg/ELP/Rapa nanomicelles. The Ki-67 assay indicated markedly reduced detection of Ki-67 protein (brown colour), which is a cellular marker for proliferation [87,88], in the tumors treated with the CCM/IR780@Arg/ELP/Rapa nanomicelles. These data unambiguously demonstrate the excellent in vivo antitumor efficacy of the CCM/IR780@Arg/ELP/Rapa nanomicelles upon NIR light. This superior tumor inhibition performance of the CCM/IR780@Arg/ELP/Rapa nanoparticles arise from the following factors: i) tumor accumulation via active targeting; ii) the CCM coating-mediated anchoring of IR780 to tumor cell membrane and cytoplasmic delivery of payloads; iii) PDT triggered by IR780 upon NIR irradiation, enabling generation of ROS that damages the tumor cell membrane as well as catalyzes L-Arg into antitumor NO gas; iv) GSH-responsive disassembly of the ELP/Rapa micelles within tumor cells, enabling efficient rapamycin release for chemotherapy.

## 4 Conclusion

In summary, a cancer cell membrane-coated ELP-based nanomicelle, which can encapsulate photosensitizer IR780, L-Arg, and chemotherapeutic drug Rapa, was designed for breast cancer treatment. The MTS-2-MTS crosslinking imparted excellent stability to ELP during storage and in vivo delivery, while enabling efficient decrosslinking in the presence of intracellular GSH. The resultant CCM/IR780@Arg/ELP/Rapa nanomicelles efficiently targeted cancer tissues and utilized a membrane fusion mechanism to anchor IR780 to cancer cell membranes while releasing L-Arg and ELP/Rapa cores into the cytoplasm. Under NIR irradiation, IR780-mediated PDT generated ROS that directly damaged the cell membrane and catalyzed L-Arg conversion to NO gas, inducing mitochondrial dysfunction. Concurrently, excess intracellular glutathione (GSH) cleaved disulfide bonds in the ELP/Rapa corona, enabling efficient rapamycin release for chemotherapy. These synergistic effects endow the CCM/IR780@Arg/ELP/Rapa nanomicelles with potent in vivo antitumor efficacy, providing insights for the rational design of cell membrane-based delivery systems.

## CRediT authorship contribution statement

**Nan Li:** Writing – original draft, Investigation, Formal analysis, Data curation, Conceptualization. **Fengyun Xu:** Supervision, Software. **Wei Zhang:** Writing – review & editing, Project administration, Methodology, Investigation. **Wenke Zhang:** Writing – review & editing, Validation, Project administration.

## Declaration of competing interest

The authors declare that they have no known competing financial interests or personal relationships that could have appeared to influence the work reported in this paper.

## Data Availability

Data will be made available on request.
