# Supplementary material for: Bioinspired membrane-fusogenic nanomicelles for synergistic chemotherapy, photodynamic therapy, and gas therapy of breast cancer
Source: Mater Today Bio. 2025 Dec 13;36:102681. doi: 10.1016/j.mtbio.2025.102681 (PMC12775932; doi:10.1016/j.mtbio.2025.102681)
Supplement: Multimedia component 1 [file mmc1.docx]

**Supporting Information**

**Bioinspired Membrane-Fusogenic Nanomicelles for Synergistic Chemotherapy, Photodynamic Therapy, and Gas Therapy of Breast Cancer**

*Nan Li^a,b^, Fengyun Xu^a,b^, Wei Zhang^a,b,^*, and Wenke Zhang^a,b,^**

^a^ State Key Laboratory of Supramolecular Structure and Materials, College of Chemistry, Jilin University, Changchun, 130012, P. R. China

^b^ Center for Supramolecular Chemical Biology, College of Chemistry, Jilin University, Changchun, 130012, P. R. China

* Corresponding author.

E-mail: [zhangwei061@jlu.edu.cn](mailto:zhangwei061@jlu.edu.cn), [zhangwk@jlu.edu.cn](mailto:zhangwk@jlu.edu.cn)

**Table S1** The amino acid sequence of ELP.

| Peptide Name | Amino Acid Sequence |
| --- | --- |
| ELP | G(VPGIG)_48_(VPGSG)_48_Y-GGKKGGKKGGCC |

**Table S2** The L-Arg encapsulation efficiency of the CCM@Arg/ELP nanomicelles.

|  | Loading rate/% |
| --- | --- |
| 1 | 23.3 |
| 2 | 20.8 |
| 3 | 22.9 |


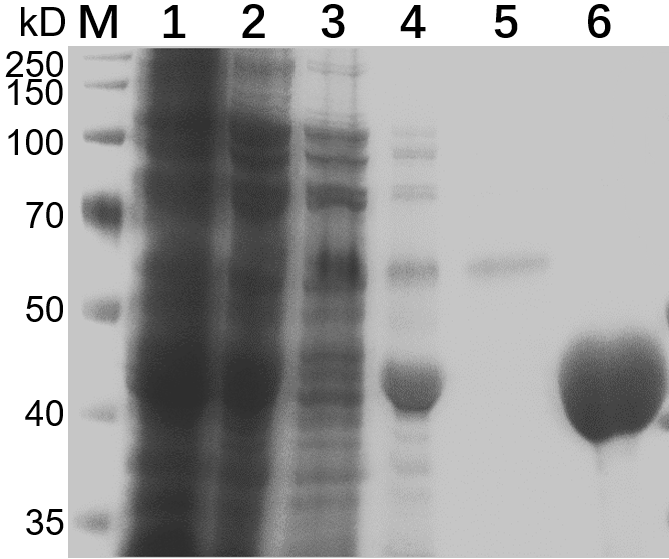


**Fig. S1.** Puriﬁcation of diblock ELPs from *E. coli* cell culture by inverse phase transition cycling (ITC) (lane M: molecular weight marker; lane 1: supernatant after sonication; lane 2: supernatant after PEI addition and centrifugation; lane 3: supernatant after hot spin during the first round of ITC; lane 4: supernatant after cold spin during the first round of ITC; lane 5: supernatant after hot spin during the second round of ITC; lane 6: purified ELP).





**Fig. S2.** The hydrodynamic diameters of the ELP micelles prepared through the thin-film hydration and spontaneous self-assembly methods.

**Fig. S3.** Drug release profiles of the CCM@Arg/ELP/Rapa and ELP/Rapa nanomicelles.





**Fig. S4.** The colocalization analysis of the ELP-Cy5 (red) with lysosomes (green) in the MCF-7 cells incubated with the ELP-Cy5 micelles or CCM@Arg/ELP-Cy5 nanomicelles (Scale bar is 10 μm).





**Fig. S5.** (a) CLSM images and (b) semi-quantitative fluorescence intensity analysis of the effect of endocytic inhibitors on the cellular uptake of ELP-Cy5 micelles and CCM@Arg/ELP-Cy5 nanomicelles in MCF-7 cells. (Scale bar is 10 μm).





**Fig. S6**. Characterization of the interaction of MCF-7 cell membranes with NBD labeled CCM (CCM-NBD), NBD labeled membrane fusogenic liposome (MFL-NBD, DMPC: DSPE-PEG: DOTAP: DSPE-NBD = 76.15: 3.85: 20: 1 (molar ratios)), or free NBD (Scale bar is 20 μm) by flow cytometry (a) and CLSM imaging (b).





**Fig. S7.** Microscopy images of MCF-7 cells after incubation with ELP-Cy5 micelles or CCM@Arg/ELP-Cy5 nanoparticles for 0.5, 1, 2, and 4 h (Scale bar is 15 μm).





**Fig. S8.** The UV spectra of the CCM/IR780 (a) and the standard calibration curve of free IR780 (b).





**Fig. S9.** The colocalization analysis of the IR780 (red) with Dil stained cell membranes (green) in the MCF-7 cells incubated with free IR780 or CCM/IR780 nanomicelles (Scale bar is 10 μm).





**Fig. S10.** Distribution of IR780 in MCF-7 cells. Cells were incubated with CCM/IR780 nanomicelles for 2 h. After removal of non-internalized particles, the culture was continued for an additional 4 h prior to imaging (Scale bar is 10 μm).

**

**

**Fig. S11.** The colocalization analysis of the Cy5 (red) with mitochondria (green) in the MCF-7 cells incubated with the ELP-Cy5 micelles, CCM@Arg/ELP-Cy5 or CCM@Arg/ELP-Cy5/Rapa nanomicelles (Scale bar is 10 μm).

(a)

(b)

**Fig. S12.** Cell viability of MCF-7 cells treated with various concentrations of the ELP nanomicelles (a) or CCM (b) components.





**Fig. S13.** Ex vivo fluorescence (a) and bright field (b and c) images of tumor tissues from mice at 24 h post-administration of the CCM@Arg/ELP-ICG, ELP-ICG, or free ICG (N=5 in each group).


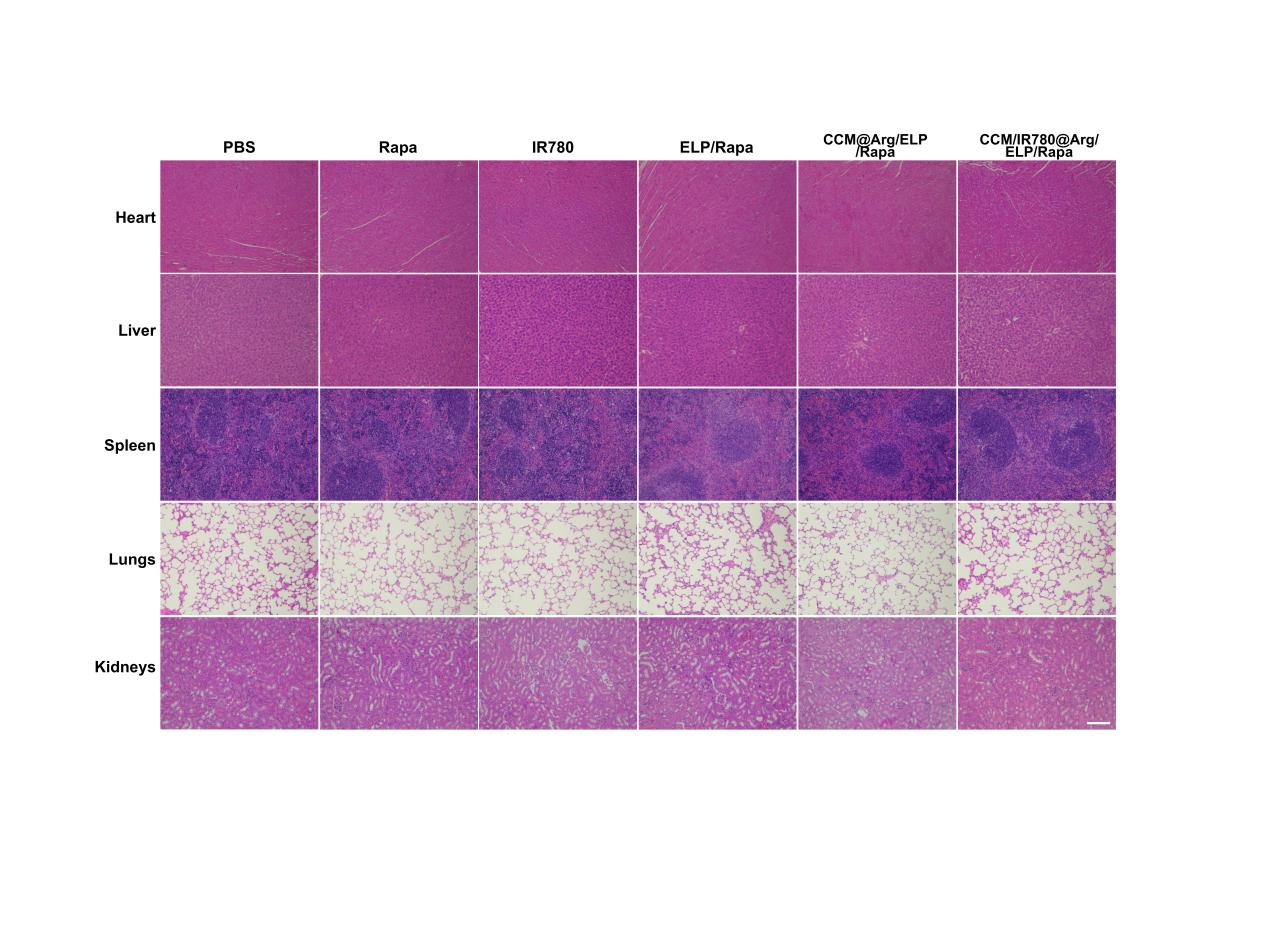


**Fig. S14.** H&E staining of heart, liver, spleen, lungs, and kidneys tissues in mice treated with nanomicelles or free compounds on day 14 (Scale bar is 100 μm).


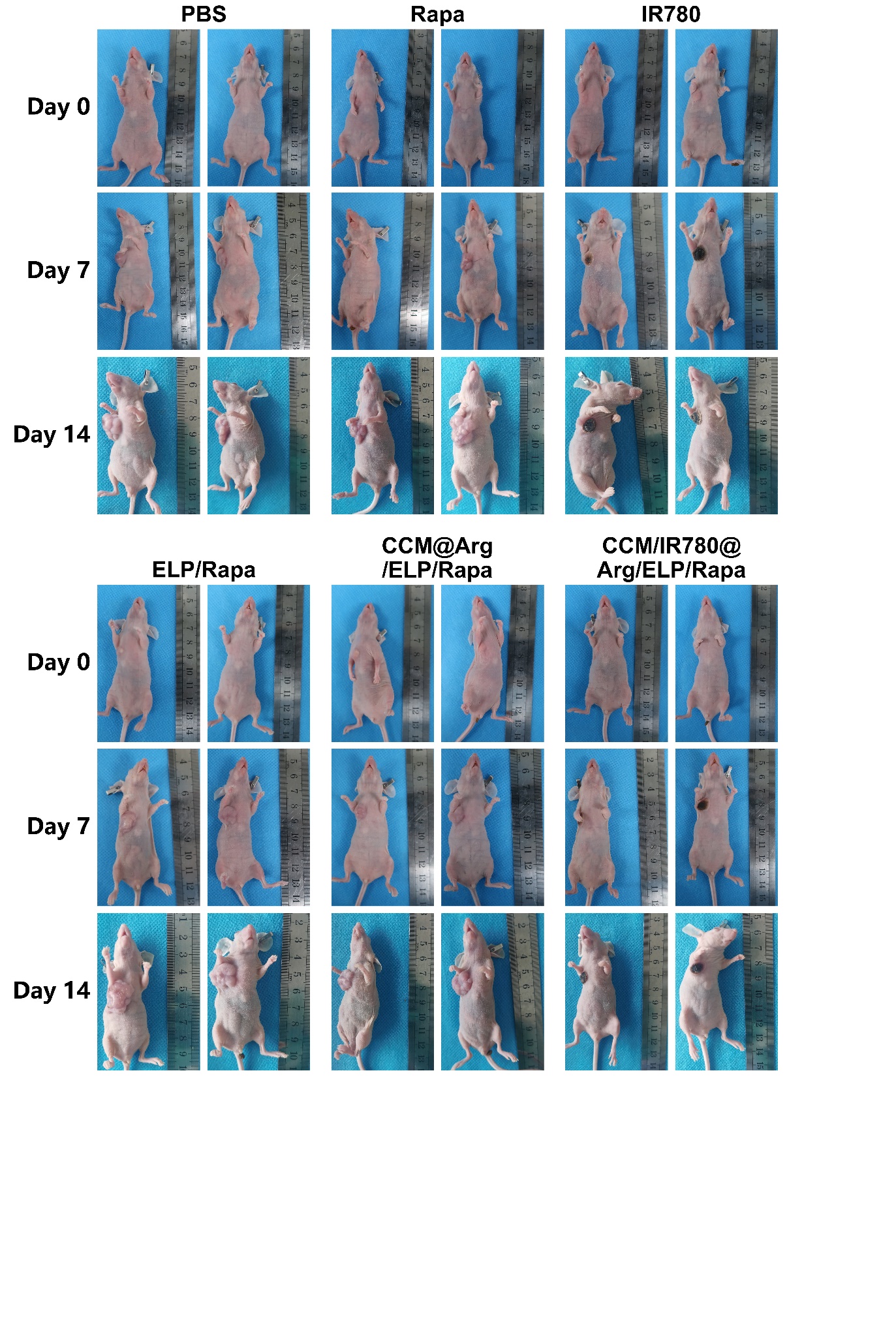


**Fig. S15.** Tumor tissues in mice treated with nanomicelles or free compounds on day 0, 7, and 14.
